# Lung cancer screening in rural primary care practices in Colorado: time for a more team-based approach?

**DOI:** 10.1186/s12875-023-02003-x

**Published:** 2023-03-03

**Authors:** Rebekah Gomes, Andrea Nederveld, Russell E. Glasgow, Jamie L. Studts, Jodi Summers Holtrop

**Affiliations:** 1 University of Colorado Adult & Child Center for Outcomes Research & Delivery Science (ACCORDS), Aurora, CO USA; 2 Department of Family Medicine, University of Colorado School of Medicine, Mail Stop F496, 12631 E. 17Th Ave, Aurora, CO 80045 USA; 3 Department of Medicine, Division of Medical Oncology, and University of Colorado Cancer Center, University of Colorado School of Medicine, Aurora, CO USA

**Keywords:** Rural population, Lung neoplasms, Early detection of cancer, Primary health care

## Abstract

**Background:**

Despite lung cancer being a leading cause of death in the United States and lung cancer screening (LCS) being a recommended service, many patients eligible for screening do not receive it. Research is needed to understand the challenges with implementing LCS in different settings. This study investigated multiple practice members and patient perspectives impacting rural primary care practices related to LCS uptake by eligible patients.

**Methods:**

This qualitative study involved primary care practice members in multiple roles (clinicians *n* = 9, clinical staff *n* = 12 and administrators *n* = 5) and their patients (*n* = 19) from 9 practices including federally qualified and rural health centers (*n* = 3), health system owned (*n* = 4) and private practices (*n* = 2). Interviews were conducted regarding the importance of and ability to complete the steps that may result in a patient receiving LCS. Data were analyzed using a thematic analysis with immersion crystallization then organized using the RE-AIM implementation science framework to illuminate and organize implementation issues.

**Results:**

Although all groups endorsed the importance of LCS, all also struggled with implementation challenges. Since assessing smoking history is part of the process to identify eligibility for LCS, we asked about these processes. We found that smoking assessment and assistance (including referral to services) were routine in the practices, but other steps in the LCS portion of determining eligibility and offering LCS were not. Lack of knowledge about screening and coverage, patient stigma, and resistance and practical considerations such as distance to LCS testing facilities complicated completion of LCS compared to screening for other types of cancer.

**Conclusions:**

Limited uptake of LCS results from a range of multiple interacting factors that cumulatively affect consistency and quality of implementation at the practice level. Future research should consider team-based approaches to conduct of LCS eligibility and shared decision making.

## Background

Lung cancer is the leading cause of cancer-related death in both men and women in the United States (U.S.), averaging around 150,000 deaths per year [1–3]. The American Cancer Society estimated the number of new cases in 2020 was 228,820 and estimated the number of deaths at 135,720 [4]. Rural areas are disproportionately affected by smoking and incidences of lung cancer in comparison to urban areas [5]. Americans living in rural areas are more likely to die from lung cancer than their urban counterparts with a 20% higher lung cancer mortality rate [6]. The U.S. Preventive Services Task Force (USPSTF) recommends patients ages 50–80 with a 20 pack-years smoking history who currently smoke or quit within the past 15 years receive low-dose computed tomography (LDCT) lung cancer screening (LCS) annually [7, 8].

In the United States, primary care practices have not widely implemented these guidelines, especially in comparison to other evidence-based screening guidelines for other cancers [9, 10]. Of the LCS eligible Americans, only 5% to 6% have been screened as of 2020 [11, 12]. While utilization varies significantly across states, people who are uninsured are less likely to undergo LCS [13]. LCS is higher among those with chronic respiratory conditions, who were divorced, separated, or widowed, who had previous cancer diagnoses, and aged 65 to 74 [13].

Various barriers to LCS exist for rural patients, clinicians, and health care facilities [14]. Rural patients who are most in need of LCS tend to have lower levels of formal education, are less trusting of doctors and health care, have inadequate insurance coverage, and face geographic access barriers [15, 16]. Rural patients also are more likely to have limited access to primary care physicians who address the LCS recommendation and referral, and to specialty care [3, 10]. LCS via LDCT in the U.S. is a covered test by Medicare and some Medicaid programs (CMS); however, Medicaid in many states does not cover it, and some state Medicaid programs do not cover it as well as some private insurances in the U.S [17]. Around 50% of those eligible for LDCT per the recommendations of USPSTF are uninsured or Medicaid insured [18, 19].

Compared with clinicians of other specialties, primary care clinicians serve an important role of ensuring appropriate screenings are recommended to eligible patients [20]. Studies of primary care clinicians have reported barriers including lack of knowledge of the current guidelines (e.g., incorrectly ordering chest X-rays over LDCT scans), implicit biases based on sex, race, ethnicity, and smoking history that hinder recommendations for LCS to patients, and lack of time, shared decision-making tools, or ability to facilitate an effective conversation on LCS [10, 21]. Disparities in access to both information and screening facilities is more severe for rural than urban patients [10].

Despite all that is known about issues related to LCS, we still need to understand more about how perspectives of different key players (e.g., physicians, other staff, patients) may influence different aspects of implementation as it relates to the context of rural primary care. With many processes in primary care, especially around cancer screenings, clinical and administrative staff play a key role in identifying eligible patients, supporting the clinician in shared decision making about the screening choice, and coordinating screening activities with providers of services and reimbursement with payers. Thus, their perspective is an important one to capture. Likewise, understanding the patient’s perspective adds insight on why patients may or may not agree to the LDCT or follow through with test completion.

We have previously used the RE-AIM framework and more recently its contextual expansion to PRISM in our implementation research [22] and determined that it might provide unique insights into different aspects of implementation, especially across the various roles of important members in the system. RE-AIM is an acronym for reach and effectiveness at the patient level, and adoption, implementation and maintenance at the setting level [23, 24]. Examining multiple key players’ views through a RE-AIM lens might reveal more about how LCS eligibility and referral for services can be conducted in rural primary care. Thus, the purpose of this paper is to understand issues related to performance of LCS activities in rural primary care practices in Colorado. We assessed the conduct of various aspects of LCS including patient identification, tracking, shared decision making, use of decision aids, smoking cessation advice and counseling, referral and follow-up from the perspectives of clinicians, practice staff, and patients and using the RE-AIM model to elucidate these issues in the hopes of overcoming potential barriers to LCS implementation in practice.

## Methods

We conducted this qualitative study as part of the Colorado Implementation Science Center for Cancer Control (https://coisc3.org); the study was approved by the Colorado Multiple Institutional Review Board (COMIRB) for research with human subjects (COMIRB #: 19–1706; date: April 27, 2020). We used methods in accordance with relevant guidelines and regulations. Recruitment occurred June – November, 2020; interviews occurred July – December, 2020. Analysis occurred throughout the interview period and ended June, 2021.

### Participants and recruitment

The goal was to elicit viewpoints from multiple important partners; thus, we asked to participate clinicians (physicians, nurse practitioners, physician assistants), clinical staff (nurses, care managers, medical assistants), and administrative staff (practice managers, front desk staff), generally three to four participants per practice. Additionally, practices recruited three to five patients who met specific inclusion criteria. Patients were to have currently or previously smoked cigarettes and be 50–80 years of age (as part of the qualification for LCS eligibility).

To recruit practices, study staff worked with the directors and staff of the State Networks of Ambulatory Practices and Partners (SNOCAP) and their member practice-based research networks (PBRNs): High Plains Research Network (HPRN), Colorado Research Network (CaReNet) and Partners Engaged in Achieving Change in Health Network (PEACHnet). In addition to rural location, purposeful selection also included a mix of ownership (federally qualified health centers versus privately owned versus health system owned), geographic location (across Colorado, U.S.) and practice size. Rural location was defined as located in a county with a rural or frontier designation or providing care for a significant number of patients residing in rural areas. We contacted 28 practices, and nine participated; many declined due to stressors from the COVID-19 pandemic but more specific information about declines was not available. All participants provided verbal informed consent per the protocol approved by the institutional review board. Recruitment continued with practices while data were analyzed until sufficient thematic saturation was achieved.

### Instruments and data collection

Interviews were conducted using a semi-structured interview guide developed by the research team to explore current practices for LCS in this setting [25]. The interview contained two parts: 1) exploration of participants’ values in a general sense – what is important to them and what brings meaning to their lives and work (reported elsewhere [26]), and 2) how the practice approached and conducted LCS and smoking cessation, including values and priority assigned to different types of cancer screening (this paper). The goal was to explore general values as well as values and importance as applied to a specific health care delivery topic (which was LCS). This second portion contained questions to explore if and how clinicians and practices currently assess smoking status, provide assistance with smoking cessation, understand LCS screening guidelines, conduct shared decision making for the LCS decision, and refer and coordinate LCS for eligible patients. We included smoking use and status because it is part of the recommendations for LCS. Interviewers used depth questioning to clarify details of the process including who did what, when, for what patients.

**Fig. 1:**
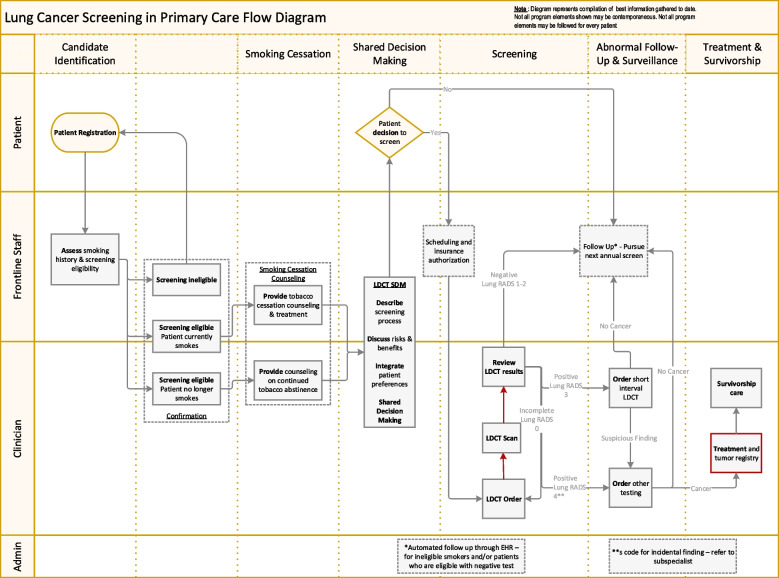
Lung cancer screening clinical work flow diagram

We also used a pre-developed work process flow diagram (see Fig. 1) to depict how LCS eligibility, screening, decision making, referral and treatment coordination (if needed), and follow-up might happen. Practice participants commented on their processes in relation to this diagram, if they did/did not do certain steps, how they ordered LDCT, and what influenced their processes. In an online shared document interviewers annotated the flow diagram (i.e., made changes in real time to the document as well as comments).

For the patient interviews, a flow diagram was not used because they were not privy to the practice’s processes for this aspect of care. Patients were asked if they had participated in or had been recommended to have LCS, if they had been asked about their smoking status and were encouraged to quit smoking, and if they received assistance with smoking cessation. The interviewers explored patients’ perspectives on these topics including importance, confidence, and barriers associated with participation.

Interviews lasted approximately one hour each and were conducted either by one or both of the qualitative analysts (R Gomes or JSH). Interviewers took extensive notes during the interviews and completed a summary immediately following the interview. Due to the COVID-19 pandemic, all interviews were done either by video or telephone. Interviews were recorded and professionally transcribed verbatim. Each participant was compensated with a $100 gift card for completion of the interview.

### Data analysis

Two qualitative analysts (R Gomes and JSH) coded and analyzed the data. ATLAS.ti version 8 (ATLAS.ti GmbH, Berlin, Germany) was utilized for data management and coding purposes. No specific qualitative philosophical frameworks were used and instead followed a thematic analysis perspective. In general, we used an immersion crystallization approach to examine the data across multiple passes and from multiple perspectives to triangulate across the researchers completing the work, the question/code categories, the respondent roles and the key features of the responses [27]. In addition, we developed and used a manual code book to code the 49 transcripts. Quotation reports were created and reviewed in discussion to identify key themes and illustrate process descriptions. Coding and analysis was inductive except for the application of the RE-AIM dimensions application to the data.

Part of the coding was driven by the RE-AIM model to capture how the LCS discussions described processes likely to influence the reach and effectiveness of their efforts as well as the degree of adoption, implementation, and maintenance at the practice level. Table 1 describes the RE-AIM dimensions and how they were defined for this study. As they reviewed the quotation reports, the analysts created a summary table to identify how different participant groups reported on the RE-AIM dimensions for the process of LCS identification and facilitation. Additionally, the analysts reviewed the annotated workflow process diagrams to glean any additional insights not found in the transcripts to add to the understanding of process elements informing RE-AIM outcomes. For purposes of this analysis, discussion of smoking status, recommendations to quit smoking, and smoking cessation were considered both part of the LCS primary care process, as well as a specific set process for assistance with stopping tobacco use. Thus, we included in the analysis pertinent statements relating to smoking and possible cessation processes. The analysts iteratively shared results with the larger research team for review and consultation. We also reviewed the relevant literature in consideration of the findings to corroborate themes as well as to consider the data from multiple perspectives.

**Table 1:** RE-AIM dimensions and definitions for this study

**RE-AIM Dimension**	**Definition for this Study**
Reach	The absolute number, proportion, and representativeness of primary care patients who are willing to participate in lung cancer screening (LCS)^a^ and reasons why or why not. Any discussion regarding the presence of absence of LCS being available to patients, which types of patients, and the factors affecting access to and use by patients
Effectiveness	The impact of getting screened for or having LCS on patient health and other outcomes, including quality of life and economic outcomes, as well as potential negative effects. Any discussion of how LCS impacted the patient or differences across different subgroups of patients
Adoption	The absolute number, proportion, and representativeness of a) settings and b) clinicians and staff who offer LCS to patients. Any discussion of the setting or people involved in making LCS available to patients, and the factors involved in making uptake of LCS provision to patients possible
Implementation	At the practice level, implementation refers to the clinicians and staff who provide LCS and their fidelity to the key elements to providing LCS and how they work. This includes a) completeness and consistency of delivery as intended, 2) the time and cost of delivering LCS, and 3) adaptations made to LCS and implementation strategies to make it happen. Any discussion of these factors including how patients experienced being offered (or not) LCS or its components
Maintenance	At the setting level, the extent to which LCS has become (or not) institutionalized or part of the routine organizational practices and policies. It also applies to the extent in which the patient receives regular (annual) LCS. Any discussion about continuing LCS as a regular practice and factors that influence that continuance

^a^LCS includes the process of identifying patients’ smoking status and eligibility for LCS, interest in quitting, and offering assistance with quitting, as well as providing shared decision making about getting LCS, completing annual testing and coordinating referral and follow-up care

## Results

Table 2 provides the descriptive characteristics of the nine practices that participated. Thirty-two patients were recruited and 23 completed an interview. Most were female (*n* = 19; 82.6%), all were White race (predominant in rural Colorado), and some reported Hispanic ethnicity (*n* = 4; 17.3%). Patients ranged in age from 53–74 (mean of 64.3) years. About half currently smoked (*n* = 13; 56.6%) with the rest reported a history of smoking. Among individuals who previously smoked (*n* = 10), about half had quit within the past year (*n* = 6; 26%). About half (*n* = 12; 51.7%) recalled being told about LCS (with about half of those deciding to undergo LCS). All were eligible for LCS based on recruitment guidelines for LCS age and smoking history eligibility.

**Table 2:** Practice characteristics

Characteristic	N (%)
Practice size	
• Small (1–2 clinicians)	4 (44.4%)
• Medium (3–6 clinicians)	5 (55.6%)
• Large (7 or more clinicians)	0 (0%)
Location in Colorado (all rural)	
• Eastern	2 (22.2%)
• South Central	3 (33.3%)
• Western	4 (44.4%)
Ownership	
• Federally Qualified Health Center	2 (22.2%)
• Rural Health Center	1 (11.1%)
• Hospital/system	4 (44.4%)
• Private	2 (22.2%)
Types of participants across practices:	
• Clinicians	9 (34.6%)
• Clinical staff	12 (46%)
• Administrative staff	5 (19%)

### Thematic results by role groups and RE-AIM dimensions

In addition to examining thematic results overall, we inspected responses by respondent group. Table 3 outlines the major thematic elements by each RE-AIM dimension. Concordance and discordance across roles is highlighted. Table 4 includes salient quotations from participants that highlight main themes from the different groups.

**Table 3:** Concordance and discordance of perspectives on Lung Cancer Screening (LCS)^a^ by Role: Practice Member (Clinicians, Clinical Staff, Administrators) and patient in rural primary care using RE-AIM Dimensions

**RE-AIM Dimension: Reach**	
** Concordance of perspectives across roles**	Insurance – Consistent issue of insurance coverage as a perceived barrier to patients completing LCS • Lack of private insurance coverage and also meeting the deductible are problems • Patients can’t or don’t want to pay for it when not covered
	Hassle – consistent views of hassles involved • For patients the time, distance, doing the driving and navigating, not wanting to miss work • For practice members the time and rigmarole involved in coordinating, getting reimbursed
	Patient resistance – consistent discussion of patient reasons for declining • Some patients have fatalistic view and are not amenable to screening; some fear and do not want to know the results; some think “it’s none of my doc’s business”, some disregard the known risks; some are amendable to screening
** Discordance of perspectives across roles**	Consistency of offering the screening – variable across groups on how often LCS is offered • Patients variable about recalling being offered or not offered LCS • Across roles and practices, variability in offering LCS from not at all, to always when eligible to occasionally
**RE-AIM Dimension: Effectiveness**	
** Concordance of perspectives across roles**	Smoking cessation versus LCS – More of the discussion about effectiveness was in the smoking cessation realm rather than LCS specifically • A few patients described the smoking cessation counseling conversation as effective with helping them quit; the provision of smoking cessation methods was helpful: (Chantix, Colorado quit line) or the way clinicians approached the conversation (“floated in the back of my mind”; doctors telling them straight forward that if they did not quit they would die) Relevance – Across groups, not many people knew patients with LC and less able to describe its effectiveness
** Discordance of perspectives across roles**	Importance – • Discordant views about screening from staff/clinicians as opposed to patients – all clinicians staff thought it important and most thought it as important as other screenings, whereas only some patients felt this way
**RE-AIM Dimension: Adoption**	
** Concordance of perspectives across roles**	Smoking cessation – • Procedures for asking about and offering smoking cessation were consistently offered as reported by all groups • Patients receive smoking cessation counseling and methods from their doctors. The only instance where this did not occur was the patient withheld his/her smoking history from the doctor or had quit prior to joining the practice
	Knowledge about LCS – • Consistent across roles describing the variability with clinician knowledge of LCS and use of CT vs. LDCT vs. chest x-rays; variable knowledge about radiation concern with every year testing; one clinician not familiar with guidelines at all
	Workflow for LCS – • Systems set up to make it easier (like EMR prompts, tickler, etc.) are a factor, variable use in practice • Some patients had been told about LCS and received the screening. Some refused the screening. Most patients that had not been told about LCS, and most of these patients were interested in learning more
	Patient factors influence clinician and team willingness to do this (burden for benefit equation) • Practice members relay that patients push back due to lacking insurance coverage, hassles and other resistance which makes clinicians less likely to want to offer it
** Discordance of perspectives across roles**	Workflow for LCS – • Clinicians most informed about why they are or are not doing this because it is falls within their role, other roles not as clear what happens with the clinician Patients had less to say about influences on adoption but were able to report whether they had been offered these things or not
**RE-AIM Dimension: Implementation**	
** Concordance of perspectives across roles**	Smoking assessment and cessation assistance – • Practice members discuss consistency of providing and how it works well, the need to be sensitive and respect patient decision • Patients described that doctors should bring up smoking cessation with patients, encouraging patients to quit but not “pushing it.”
	Communication – • Practice members communicating without being condescending with patients echoing similar sentiments
	Knowledge about shared decision making with LCS – • Most clinicians are not doing shared decision making as they describe it (say they are but are not by description); some gaps for some in knowing about this mandate and other guidelines for LCS • Some practices: shared decision making is employed to get the patient to “say yes”; some patients confused about being billed for telehealth since not in the office
	LCS Work flows – • LCS doesn’t get done as much as other screenings because there are more guidelines and criteria to figure out as well as steps to do; unique from other screenings • Lack of time is a factor (many other issues, not getting paid when patient not there) • Telehealth has made figuring out patient issues easier; portal helps communication for smoking cessation; Follow-up on smoking cessation lacking – one-time conversation; inconsistency of recommendation by clinician for smoking cessation (training might help); having a regular MA/Dr pairing may facilitate more efficiency (patient doesn’t have to repeat the spiel); LCS being done with wellness visit or other types of visits
** Discordance of perspectives across roles**	LCS Work flows – • Clinicians and staff much more on how to make it happen consistently and well in the practice • Patients who had it done reported being asked and having follow-up and recommendations
	Quality metrics/reporting—quality report does not have LCS on it right now; there are quality metrics for many screenings—is there for this?
**RE-AIM Dimension: Maintenance**	
** Discordance of perspectives across roles**	Maintenance was covered less as a topic than other RE-AIM dimensions overall
	At the patient level—was considered important for some patients to continue to stay quit and get LCS as recommended; others not so much for reasons covered in other categories
	At the practice level—Clinicians recognize the need to do annual screening but there are implementation issues with doing so • Hard to recommend annually (concern for radiation risk to patients) and just plain remembering to do it again and where patient is in the process

^a^Discussion refers only some parts of the process of implementation LCS (i.e., identification of eligibility, conducting shared decision making or recommending LCS, and having patients get testing for LC). Smoking and smoking cessation parts of the LCS process are noted separately

**Table 4:** Illustrative quotations demonstrating themes by RE-AIM domain

**Participant role – Theme **	**Quotations **
**Reach:** *Illustrating the hassles involved with LCS across groups or anticipated resistance*	
Clinicians/ Provider – LCS	“Most people are pretty open to it…We always have a few that are like, “I don’t wanna know.” But most do follow through for the appointment, yes.” [MD1 60004] “I think the two biggest impediments I see to people getting that done are, well, probably three things: one is cost; one is difficulty of getting there, and spending a whole day coming and goin’; and the third thing is, you know, a lot of ‘em say, “Well, if I get lung cancer, it’s my time. I’ll go, you know. I’ll take my chances.” [Laughs – 26.17]. “If God wants me to go, I’ll go.”” [MD1 60003]
Clinical Staff – LCS	“You know, with the Obama care, the colorectal, the mammogram, the pap, the prostate, annuals, all those are covered on your insurance by law. All insurances have to cover those screens, but the lung cancer’s still not on there. The last time I tried to get an authorization on any of the insurances except for Medicare, 65 and older… it’s just a wall” [PN1 60001]
Clinical Staff – SC	“A lot of information given to the patients, really, and because we’re so small, the provider, she’s got everything laid out and ready to go. So, [laughing] it’s a matter of just going and grabbin’ it, and handing it to the patient, or you know, we do a lot of—our records are kept through electronic health records. And so, a lot of communicating that way sometimes, also is done as far as providing information.” [MA1 60004]
Administrators – LCS	“I’m not positive because, again, I’m not in that part of the EMR, but I think they send them to Community, or depending on their insurance, over to St. Mary’s. But I’m not sure ‘cause I have never ordered one.” [AD1 60002]
Administrators – SC	“I guess once they say, “Yes. I smoke. No. I don’t wanna quit. I don’t even wanna hear anything about it.” Then, again, it’s just personal, like, what can you do as to the physician. I mean, again, he can tell them anything they want and screen ‘em and give ‘em options, but it’s the person that needs to do the change.” [PM1 51105]
Patient – LCS - Not willing to address	“It’s like sticking your head in the sand.” [P2 70002]
Patient – LCS - For screening	“Yeah. It’d be nice to know if it was there. I mean especially I smoked so long. Again, I mean it would be nice to know. If I have lung cancer, I don’t know what I would do about it at this age. I mean I’m not sure which way I’d go on it, but yeah, it’d be nice to know.” [P2 60001]
Patient – LCS - Against screening	“[Y]es, I smoke. I know that’s not good, but I’ve never had… any problems that makes me think, oh, I guess I better go get this checked out… Well, I think that –it is something I think that in my generation…you just didn’t go to the doctor just because of this or that. And I am still kind of one of those that, oh, let’s just give it a while and see if it gets better on its own.” [P3 51401] “You know, lung screens, all those things are not paid for…And when you have $6,000 deductibles and then… we only have [a] hospital, so that means when I’ve had to have some x-rays…I had to leave town or otherwise they were gonna be $500. Where if you left town, they’re $125. So, there is a cost associated with it, and people say, “Well, how much do you pay for some cigarettes.” I mean I hear [inaudible] verse the back and forth, but you’re not talking hundreds of dollars all at one time, you know.” [P3 51401] “Well, probably ‘cause in my mind it’s when I see it, then it’s probably too late, or unfixable, or maybe it’s ‘cause I don’t wanna take the first step to stop. I’m not really sure what the fear is… Kind of don’t fix it if it ain’t broke rather than I’m thinkin’ it might be patched up right now. So, maybe, like, patched together.” [P4 60002]
Patient – SC - For SC	“Because my surgeon told me—it’s the surgeon that saved my life told me, “If you keep smoking cigarettes, you’re going to die.”… maybe I really don’t need that frickin’ cigarette… I have no desire to pick up a cigarette. Done. No cravings, no desires, none.” [P2 60002]
Patient – SC - Against SC	“I think my doctor knows that I smoke anyway. Not that I told, so I don’t think it’s any of her business. Then I wouldn’t have a life if I stopped smokin’ and drinking—a beer every now and then. If I stop smoking and drinkin’ beers, what am I gonna do? Other than watch TV already. I do that.” [P3 51105]
**Effectiveness:** *Illustrating patient versus practice member discrepancy on the importance of LCS*	
Clinicians/ Provider – LCS	“I definitely think it’s important, but I value it and look at it all as the same of all cancer screening preventative measure. And usually that’s [what I] tell every patient, we only have so many things that help to screen and prevent for cancer and might as well do ‘em… A lot of cancers that we don’t—we can’t screen for, so you know, this is one. So, take what we have resources for.” [PA1 70001]
Clinicians/ Provider – SC	“I think it would be better use of time to get ‘em to stop smoking because that pertains not only to cancer…So, you’re hitting more boxes if you get ‘em to stop smoking, I think. But that’s assuming that that intervention of talking to them about smoking cessation [is heard]. You’re odds of getting ‘em to go do screening are better than your odds of getting ‘em to quit smoking. You really ought a do both though.” [MD1 60003] “Very, very important. So, the earlier we can catch these risk factors and catch people with conditions, the better. Definitely.” [CC1 60001] “It’s usually fairly high up my list. If I have something that might be currently threatening their life…we will probably not talk about smoking cessation…But if there’s any room for any conversation about how to improve your health rather than just dealing with the most urgent acute issue, then smoking cessation is high on that list. [MD1 51105]
Clinical Staff – LCS	“The first one…he was around my age, and he was not feeling well….And we had done x-rays of the chest….And so, when we finally had done multiple imagining…something come up on a CT… And he was upset because we didn’t find that sooner…And I was in on most of those appointments, and there was never anything like, “I can’t get rid of this cough,” [I]t wasn’t something you’d just say, we outta check your lungs. He was a smoker, so this 30-pack, you know, all the guidelines for the lung cancer screening probably would have got him in quicker for a lung screen. But anyway, he ended up with small-cell lung cancer…but he did pass away from that…and it was very sad. [PN1 60001]
Clinical Staff—SC	“I know they do the discuss risks and benefits. And other than that—I just feel like they’re just referred out to quit line and stuff like that, unless they’re going to start taking medication, then it’s kind of like they’re prescribed medication, and then it’s like, okay. We’ll follow up, and then they come back and follow up, and then just kind of go that route, but other than that, I don’t feel like there’s much done about it.” [MA1 51105]
Administrator – LCS	“I think it’s probably pretty important. I’m not sure that we remember to do it all the time. But I do think at least, at the very least, asking the questions about risk factors, that piece of the screening is super important.” [AD1 70002]
Administrator—SC	“I think it’s like everything else. Patient needs to be aware of the risk, and sometimes it’s anything just like any other disease. I think it’s also cultural, you know, well, before people—it was normal to smoke. So, it’s their culture to think that it’s—it’s just normal. But, yeah, it’s important just like any other disease, too, to say, you know, it’s bad, and this is what’s gonna happen, and then give ‘em risk and consequences, yes.” [PM1 51105]
Patient – LCS	“But that was a good experience, you know, just talking to my provider. And when they mentioned all this stuff about lung cancer and everything, and that’s kind of scary. But they were real encouraging, and very supportive, so that was a good thing. When you feel comfortable in the clinic talking to your PA, you know, it makes a lot a difference. And I feel better now, so yeah, that is a good experience. I feel better.” [P4 70001] “I understand I don’t need to smoke. I need to stop. I got it. But sometimes that’s easier said than done.” [P1 60004]
Patient -SC	“I just quit smoking. And they’d say, “Good for you!” You know, I mean that was an encouragement…I was excited about that because they said, “Good for you.” But they really don’t make you feel ashamed.” [P1 51401]
**Adoption:** *Illustrating practice member barriers to doing LCS*	
Clinician/ Provider – LCS	[W]e always talk about low-dose CT—LDCT. And I’m not sure how that’s different from the CT that they do when I order a CT of the chest…[the] reports always come back, “We used the lowest possible dose,”… Do they, or is low-dose CT for lung cancer screening something special that only limited number of places have? [ MD1 60003] “I just worry about is the whole radiation side of things. I’m not very good about recommending it every year. I will recommend it, but then I don’t always, like, feel super excited about in a year’s time saying, you need to go get this again. If it’s been several years, then I feel more comfortable.” [MD1 60001]
Clinician/Provider – LCS and SDM	“It’s fairly simple. I mean, so you’ve been smoking for a long time. It’s now recommended that you have screening. There’s a low-dose CAT scan that they do to look for any signs of lung cancer. It’s usually recommended yearly. Is it okay if I go ahead and send a referral for you to do that?”… I don’t have a tool, no.” [MD1 60004]
Clinician/Provider – SC	“We assess their smoking. That happens annually… [S]o it’s actually one of the quality or we call it our QI tab. So, if it’s been over a year, we get an alert that says we need to assess it. So, it’s definitely done once a year…. [I]t is on our annual questionnaire, so we have patients fill out a review of systems, basically, and it’s also one of the questions on that. So, that’s once a year, and if it’s not written down, then the clinician asks. [MD1 60002] “Well, we ask them about their smoking history at every visit, and you’ll have to check with the frontline staff as to what their protocol is as far as what they offer the patient at that point in the visit.” [MD1 60003]
Clinical Staff – LCS	“That’s something the provider does, and I’m not sure what kind of assessment they do to determine if that patient is eligible. So, that’ll have to be a conversation that, you know, with my provider.” [LPN1 70002]
Administrator –LCS	“I’m not for sure. That would be a question for one of our providers.” [AD1 70001] “So, we don’t necessarily have like a process of if they have this, then they can get this. It’s usually done by the provider, so the provider usually makes that call and puts in the order and stuff like that. It’s not usually done by the nursing staff. The provider’s the one to say, like, “Oh, yeah. They need this done.” But we don’t necessarily have like certain guidelines that the nurses go through to say, yeah, this one’s gonna need a CT low dose.” [AD1 70003]
Administrator—SC	“We usually ask people if they’re interested in quitting smoking, and we usually tell ‘em, you know, the only think smoking’s good for is cancer or heart disease, and high blood pressure. And then, if they’re not really interested at that point in time in quitting smoking, we kind of just sort of let the conversation go. If they say, “Yeah. I’m interested in it.” Then, you know, we can start talking about the options that are available.” [AD1 70002]
Patient – LCS	“No. She hasn’t ever talked to me about it. The only thing she talked to me about was quitting—to quit smoking. That was it.” [P1 70003] “It was pretty much, like you know, “Well, they have lung cancer screening now, and you could be a good candidate.” And I said no.” [P1 60002] “Umm just that she thought I should do it because I smoked for so long, my mother died of cancer, you know, lung cancer I should say, and at that time I was still smoking.” [P2 51105] “I get it once a year, I guess. But whatever it is, yeah. I know how she’s—and she’s always on top of my smoking, when I’m smoking, and my drinking. She’s on top of that too… She just wants me to be aware.” [P2 60001]

*LCS *Lung cancer screening, *SC *Smoking cession, *SDM *Shared decision making

#### Reach

Two main factors affected reach. One is the offering of LCS to patients, and the second is patients’ decision to get LCS and then complete the LDCT. We found that although the risk assessment and smoking cessation aspects of the flow diagram (Fig. 1, columns 1 and 3) were reported as happening routinely, the screening eligibility and shared decision making (columns 2 and 4) were not. Considering the rows that depict which team member conducts the activities, it is the clinicians who have often not yet routinized these actions, suggesting that smoking cessation processes are established protocols for staff whereas the LCS components are not. All groups reported that LCS was offered less consistently to patients than smoking status assessment and cessation assistance. Many respondents spoke to the desire to establish systematic processes for LCS eligibility such as requesting EMR prompts, reminders and templates such that the clinican could be ready engage in shared decision making with the patient. For the second factor (patient agreement and follow-through), represented in the actions needed in columns 4 and 5, (shared decision making and screening respectively in Fig. 1), all parties identified issues such as perceived lack of insurance coverage, hassles experienced by patients, and resistance to screening by some patients. The patients provided the most robust explanations of their resistance and the factors that influenced their unwillingness to have LCS or to quit smoking.

#### Effectiveness

For clinicians and clinical staff, LCS is consistently stated as very important, and as important as other cancers or health issues; however, these roles also reported having few patients with lung cancer, which made it appear less relevant than other cancers. In contrast, all types of practice members types emphasized the importance of smoking cessation for preventing lung cancer. Their frustration was in finding patients interested in quitting smoking and successfully encouraging those patients to consider it. The few patients who had completed LCS thought it was important and effective in preventing death from LCS; however, many others had not completed LCS and stated reasons for skepticism such as fatalism, stigma, and money making by health care entities, as well as just not wanting the hassle of another medical intervention. Some patients expressed that they should quit smoking (indicating effectiveness in improving health), but doubted they could or ever would.

#### Adoption

Factors affecting adoption are those that influence whether the clinicians and clinical team offer smoking cessation and LCS. Smoking cessation was generally offered routinely as reported by most practice members of all types. There was less consistency across groups on LCS because only the clinicians knew how to conduct this process; other groups did not know about what happens or why. This highlighted that while screening for smoking was a routinized process for staff, asking about eligibility for or interest in LCS was not. It was largely the role of the clinician to determine eligibility and offer LCS. There was expressed openness to some parts of the process being systematized by staff. Clinicians relayed that they completed the discussion utilizing principles of shared decision making, but when we asked about a typical conversation, they did not describe processes of shared decision making, and none used any shared decision-making tools or aids. Patients both did and did not recall being offered smoking cessation or LCS. The clinicians shared how patient response could make them feel less willing to offer LCS – when patients demonstrated difficulty or resistance, the clinicians were less likely to want to discuss LCS with future patients as it was perceived as a hassle. The benefit/burden of spending their time with LCS shared decision making was not always perceived as worthwhile (“a lot of burden”).

#### Implementation

For implementation, we sought to understand factors influencing what would make LCS or smoking cessation go better or worse. In general, there were more implementation challenges with shared decision making with LCS than for smoking cessation, which has been performed routinely for much longer, resulting in established and effective workflows. Patients tended to focus more on *how* the clinician or clinical team brought up smoking and whether they communicated in a way that made the patients want to quit smoking or get screened (for LCS), rather than on the process. However, clinical team members focused more on how they could institute processes to make asking and offering help more consistent, although some clinicians did also acknowledge that how the subject was brought up can affect patient receptiveness. All practice member groups endorsed that there were more problems with implementing LCS than smoking cessation because eligibility, understanding reimbursement or insurance coverage, referral coordination, and ensuring LDCT was performed is more difficult than smoking cessation. In summary, many of the steps necessary to conduct shared decision making for LCS were complicated and contingent on previous steps as well as largely reliant upon the clinician to remember to do them.

#### Maintenance

Maintenance in terms of helping patients to maintain tobacco cessation and continue to get annual LCS was discussed less often. There were also clearly implementation challenges with maintaining annual shared decision making for LCS at the practice level as comments were made about needing reminder systems for annual discussions and follow-up to check if patients did get the LCS that was recommended to them.

### Summary

Overall, we found that shared decision making for LCS often does not happen due to intersecting and perhaps interdependent factors. If just one step in the process does not happen, LCS does not happen. This includes: 1) identifying eligible patients – often performed by a clinician and not involving other staff and/or automated procedures except to identify smoking status, 2) the clinician using shared decision making to offer LCS as per the CMS mandate for payment to ensure reimbursement, 3) the patient deciding to undergo LCS, 4) office staff scheduling and coordinating LCS, 5) insurance approval leading to scheduling LDCT at another facility, 6) the patient traveling to and attending the appointment, 7) the clinician receiving and reviewing the results, and 8) the clinician or other health care professionals conducting any necessary follow-up. Like our workflow diagram in Fig. 1, there are multiple places where these steps may not occur. It became clear that with LCS, as opposed to smoking cessation, much of the work is in the clinician’s workflow rather than the staff.

**Fig. 2:**
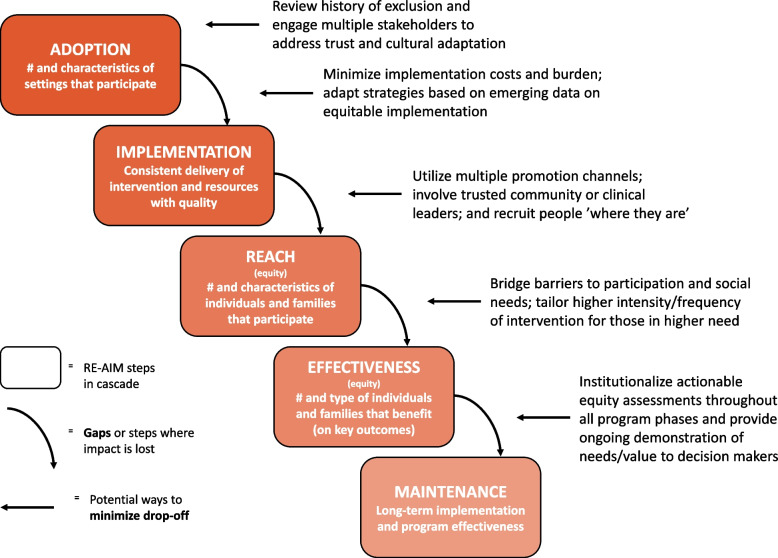
Cascade of events influencing RE-AIM outcomes

Cumulatively the above issues create a cascade effect to produce low rates of LCS. The RE-AIM analysis sheds light on why each of these steps might not happen or be implemented with quality. We used a separate flow diagram (Fig. 2) of RE-AIM to consider the typical cascade of events that results in low effectiveness of an intervention [28]. From our data in this study on the issue of LCS implementation, we found consistency with this figure. Starting from the top left of the diagram: 1) Adoption – clinicians lack the knowledge/time to properly discuss and initiate shared decision making for LCS, 2) Implementation – clinical teams and their workflows are not systematized to get LCS eligibility or shared decision making prompted for the clinician or for others on the team to do those tasks or to follow through with referrals, 3) Reach – as a result patients are not offered LCS consistently or in a way that is compelling and feasible, so they do not go, which affects 4) Effectiveness – reduces the impact and benefits of LCS for patients. Last, 5) Maintenance—some patients believe it is not something they should do or it is a hassle, and it falls off the radar, then this reinforces the practice not doing it. Considering the interconnectedness of these steps (Using RE-AIM, process diagrams or related approaches) and understanding how decisions further upstream or earlier in this cascade can affect results downstream may be useful for future interventions aimed at improving LCS in rural settings.

## Discussion

Although previous studies have examined the challenges of consistently conducting shared decision making for LCS in rural primary care, this study is unique in its use of qualitative methods, an implementation framework (RE-AIM) for examining implementation issues specifically, and assessment of multiple participants in the primary care setting including patients in order to triangulate viewpoints. Key new findings for the understanding of LCS implementation include the lack of systematic processes that integrate clinic staff for shared decision making for LCS (as compared to smoking cessation and other cancer screenings [29]), which is further complicated by contingencies based on patient responses and preferences and stigma around the relationship of smoking to lung cancer. A main recommendation is further study to investigate if systematizing the process and involving more practice team members results in better uptake of LCS.

Some of our results replicate those found in prior research about LCS in rural settings [30, 31]. These include lack of geographic access to LDCT screening programs and limited or ineffective clinician-patient communication [19, 32]. Although previous studies assessing patients’ perceptions of cancer screening found that fatalistic belief systems contribute to patients’ declining LCS [33], this factor seemed especially strong among patients in our study.

Further research is needed to investigate what might be done to overcome this challenge, but it may be due in part to the combination of long term smoking, stigma regarding smoking [34, 35], sociocultural beliefs about lung cancer having a poor prognosis [36, 37], and social determinants of health challenges [38, 39] since both rural patients and people who smoke tend to have more social needs challenges than other groups [40, 41].

As with other research [39, 42] there was also confusion related to insurance coverage and costs associated with LCS. These perceptions run contrary to established policies and regulations dictating coverage for Medicare beneficiaries, Affordable Care Act policies given the USPSTF B grade for service recommendation, and coverage of LCS for Medicaid beneficiaries in Colorado [43, 44]. This barrier was identified following the initial launch of LCS while payers adapted their coverage and data management systems, but issues have substantially declined with more years of experience. It is important to explore this barrier more to determine if insurance issues are linked with inappropriate coverage refusals by payers or if initial coverage challenges contributed to sustained misperceptions regarding LCS policy.

This paper further contributes to the existing literature because of its focus on implementation within the practice. Of note, we found smoking identification, counseling, and referral to resources and assistance were systematic processes involving staff with follow-up support by the clinician with patient decision making; whereas, these processes for LCS were largely absent. Although the shared decision making may still rest with the clinician, other parts of the process – such as calculating pack years – could be added to staff responsibilities and be systematically collected. As found by Slatore, et al. [45] in their survey of practitioners in Oregon, the process was essentially left up to the clinician. They also found that registry and EMR systems lacked support for this effort [45]. This was especially true for rural settings in their study. This finding aligns with research showing that a centralized LCS program may be better suited to manage annual and follow-up screening [46], while maintaining communication with referring primary care clinicians regarding patient management. Other research describes how to set up a team-based approach for other cancer screenings and recommends that the same be done for LCS [47].

In examining perspectives of different participants in this process, we found similar barriers across groups regarding the practical difficulties of completing LCS and the variable response among patients to shared decision making regarding LCS. Patients were able to explain in detail their reasoning for accepting or declining LCS. Some wanted and took the opportunity to get LCS because it might help catch cancer early and prevent death, and others had belief systems that were inconsistent with screening such as it being not possible to change (“it’s my time when it’s my time to go”) or not necessary (“deal with it when something comes up”). Shared mental models about what something is, how it works, and why it is important are an important implementation concern [48]. One potential option is to systematically elicit patient perceptions as part of the shared decision making process, such as through a pre-visit questionnaire or having the patient watch a pre-visit video [49, 50]. Knowing their perceptions would illuminate to what extent patient values and perspectives are influencing the process of getting LCS versus logistical or financial concerns and help the clinician focus their consultation time on correcting misperceptions or problem-solving barriers to screening.

We found the RE-AIM model was helpful to conceptualize and categorize factors related to LCS [51, 52]. Using RE-AIM made it evident that some dimensions were discussed less often than others; for example, practice staff paid less attention to the effectiveness of LCS and the maintenance of continuing LCS. This has significant implications for implementation and points to areas for intervention in terms of clinical consultations regarding the process and value of LCS. In particular, the cascade effect of the interaction among several sequential factors in the RE-AIM model (e.g., if a patient was identified; if so, then approached for discussion, etc.) was particularly salient.

Our findings have several implications for implementation of LCS initiatives directed at rural primary care practices and their patients. First, context is a critically important factor, which we define broadly: in addition to physical settings and available resources and workflows, there are also the more subjective contextual issues such as history and patient and clinician values. Patient perspectives and preferences may be different and require different strategies. For example, in our study, some patients voiced concerns with getting health care interventions, consistent with a minimizer perspective [53], which may be more common in rural areas [26]. Second, processes for implementing LCS in rural settings need to be pragmatic, not overly time consuming, fit into existing workflows, and tap available resources in rural primary care as opposed to those applicable in large integrated care settings where much of the research on LCS has been conducted. This may be particularly challenging in rural settings that have less access to centralized LCS programs that take a more active role in managing the full LCS process and helping patients navigate the complexities. One important workflow option could involve clinical staff assessing smoking status and offering smoking cessation, including calculating eligiblility for LCS, while clinicians maintain responsibility for the LCS discussion with the patient. Third, outcomes could be enhanced by educating staff regarding 1) current eligibility/and reimbursement details; 2) differences between screening for high-risk patients versus diagnostic follow-up of symptomatic patients; and 3) importance of implementing of high quality shared decision making and smoking cessation counseling and not just “checking the box” [54, 55]. Finally, many rural settings do not have state of the art EHR systems or other technologies; enhancing automated identification and prompting systems for identification and follow up with LCS eligible patients, and conducting ongoing audit and feedback would likely enhance success.

Limitations of this study include the relatively small sample size in Colorado, although we did find thematic saturation and feel the sample was sufficient for this qualitative exploration. Our findings need replication – especially as they were obtained during COVID-19 and at a time when policy and reimbursement issues around LCS remain poorly understood. This as well as the relatively new and somewhat changing and uncertain specifics around requirements for reimbursement might well produce findings that could vary over time. Our study also has strengths and unique contributions, especially the use of an implementation science approach to LCS with use of RE-AIM and multiple perspectives for the analysis [56].

## Conclusion

In conclusion, there are multiple contextual factors that affect implementation of LCS and performance of shared decision making in rural settings. Several of the perceived challenges were shared across different types of participants. Future research should attempt to replicate and expand our findings in different settings and evaluate interventions based on proposed recommendations regarding more robust assessment of smoking by staff and integrating additional tools to educate candidates for LCS.

## Data Availability

The data generated in this study are available upon request from the corresponding author.
